# ARAP1 fine-tunes F-actin polymerization level in lymphocytes through RhoA inhibition

**DOI:** 10.3389/fimmu.2025.1591450

**Published:** 2025-12-18

**Authors:** Yoshihiro Ueda, Naoyuki Kondo, Yuji Kamioka, Tatsuo Kinashi

**Affiliations:** Department of Molecular Genetics, Institute of Biomedical Science, Kansai Medical University, Hirakata, Osaka, Japan

**Keywords:** chemokine, RhoA, RhoGAP, RA-domain, ARAP1, F-actin polymerization

## Abstract

Rho family of small GTPases play crucial roles in F-actin polymerization and actomyosin contractility, facilitating lymphocyte polarization, motility, and adhesion. However, the spatiotemporal cooperation of these processes remains unclear. In this study, we found that the dual GTPase-activating protein (GAP) ankyrin repeat and PH domain 1 (ARAP1) modulates RhoA activity through its Ras-association (RA) domain, which binds to Rac and Rap1 and is critical for F-actin polymerization and cell migration. ARAP1 was transiently recruited to cell protrusions following chemokine stimulation. ARAP1-deficient cells exhibited enhanced chemokine-directed migration, accompanied by increased RhoA activation and F-actin polymerization. Conversely, ARAP1 overexpression had the opposite effect and inhibited migration in a manner dependent on its RhoGAP domain. Notably, the RA domain bound Rap1 and Rac1 and was required for ARAP1-mediated RhoA inhibition. These findings indicate that ARAP1 modulates RhoA activity at Rac/Rap1-rich protrusions and fine-tunes F-actin polymerization and cell motility.

## Introduction

Rho family of GTPases plays a central role in lymphocyte migration by controlling the reorganization of actin cytoskeleton. Chemokines activate Rac GTPases, which trigger branched F-actin polymerization through the WAVE regulatory complex. This process uniformly induces bursts of F-actin with multiple F-actin-rich protrusions at the cell periphery. Subsequently, F-actin is polarized to one side of most cells at the leading edge (1, 2). In contrast, RhoA promotes uropod formation at the rear in concert with the accumulation of ezrin, radixin, and moesin (3, 4). In this process, RhoA activates Rho-associated coiled-coil-containing protein kinase (ROCK), which phosphorylates the myosin light chain (MLC) to activate non-muscle myosin and drive actomyosin contractions. RhoA also directly activates the formin mDia/profilin complex to promote linear F-actin polymerization, thereby contributing to protrusion extension at the leading edges (5, 6). Overall, Rac1 and RhoA may cooperatively extend F-actin networks to generate actin-driven propulsion and facilitate migration. Guanine nucleotide exchange factors (GEFs) and GTPase-activating proteins (GAPs) play vital roles in this regulation; however, the detailed regulatory mechanisms governing lymphocyte polarization and migration remain largely unknown.

The small GTPase Rap1 is a potent activator of integrins that mediates cell adhesion and migration in response to external stimuli, such as chemokines, and induces cell polarization (2, 7). In our previous study, we showed that Rap1 transduces Rac-mediated F-actin polymerization signals into RhoA signals via GEF-H1 and talin1, thereby enabling the coordinated establishment of front-back polarity and promoting cell migration (2). However, the molecular mechanisms by which Rac and Rap1 modulate RhoA activation remain to be elucidated. Accordingly, we sought to identify the spatiotemporal regulators of RhoA downstream of Rac and Rap1.

One such candidate molecule is ArfGAP with RhoGAP domain and ankyrin repeat and PH domain 1(ARAP1), a dual Arf and RhoGAP protein that also contains a Ras/Rap1-associated (RA) domain (8). Previous studies have demonstrated the critical role of ARAP1 in F-actin dynamics and membrane transport processes. For example, ARAP1 regulates Golgi structure, the ring size of circular dorsal ruffles, and EGF receptor trafficking via its ArfGAP activity (8–12). Additionally, ARAP1 controls cell rounding and stress fiber formation in epithelial- or fibroblast-derived cells, modulates F-actin dynamics at podosomes and sealing zones in osteoclasts, and suppresses adenocarcinoma metastasis via its RhoGAP activity *in vitro* and *in vivo* (8, 9, 13, 14). Despite these findings, the regulatory functions of ARAP1 and its relationship with Rac and Rap1 in lymphocytes remain largely unknown.

Therefore, this study aimed to elucidate the role of ARAP1 in lymphocytes, focusing on F-actin dynamics. Here, we identified a novel mechanism by which ARAP1 negatively regulates chemotactic lymphocyte migration through modulation of F-actin development. Specifically, ARAP1 suppressed both F-actin polymerization and RhoA activation through its RhoGAP domain. Moreover, the RA domain of ARAP1 interacted with Rac and Rap1 and was required for ARAP1-mediated RhoA inhibition. Collectively, our findings demonstrate that ARAP1 modulates RhoA activity at Rac/Rap1-enriched cell protrusions and fine-tunes F-actin polymerization and lymphocyte motility.

## Materials and methods

### Cell lines

Ba/F3 cells (CVCL_0161) transfected with human(h) LFA-1, named Ba/F3.hLFA1, were generated by knocking out mouse ItgaL and Itgb2 using CRISPR/Cas9 technology and subsequently transfecting with hITGαL, hITGβ2, and CD62L under the promoter of human EF-α (15). Ba/F3.hLFA1 cells were maintained in complete RPMI medium containing 6% FCS and IL-3 conditioned medium.

### Animals

C57BL/6J mice were purchased from CLEA Japan and maintained under specific pathogen-free conditions in the animal facility at Kansai Medical University. H11Cas9 CRISPR/Cas9 knock-in mice (JAX: 028239) were obtained from Jackson Laboratory. All animal experiments were performed in accordance with protocols approved by the Animal Care and Use Committee of Kansai Medical University (approval no.: 25-085 (5)).

### Antibodies and reagents

Antibodies specific for Rap1 (610195), SNAP-Tag (P9310S), α-tubulin (T9026), anti-RhoA-GTP (26904), and RhoA (sc-418) were purchased from BD Bioscience, Promega, NewEast Biosciences, Sigma-Aldrich, and Santa Cruz Biotechnology, respectively. Goat anti-ARAP1 antibody was obtained from Abcam (ab5912) or Sigma-Aldrich (SAB2501827). Fluorescently labeled (FITC, PE, and APC) antibodies specific to CD44, CXCR4, and human integrin αL were purchased from BioLegend. Phalloidin conjugated with iFlour488 (ab176753), iFlour555 (ab176756), or 647 (ab176759) were purchased from Abcam. Recombinant CXCL12 (350-NS) and CCL21 were purchased from R&D Systems.

### T and B cell purification and T cell stimulation

To isolate primary T and B cells, single-cell suspensions of splenocytes from C57BL/6 mice were prepared and subjected to magnetic cell sorting using the Mojosort mouse pan T Cell or mouse pan B Cell Isolation Kit (BioLegend), according to the manufacturer’s instructions with minor modifications. Notably, the purities of T and B cells typically exceeded 95%. To obtain T cell blasts, purified T cells were stimulated with immobilized anti-CD3 (10 μg/ml) and soluble anti-CD28 antibodies (2 μg/ml) in the presence of human IL-2 (50 U) and cultured for 7 days, as previously described (15).

### RT-PCR

Total RNA was extracted from cells (5 × 10^6^) using Isogen-LS (Nippon Gene) according to the manufacturer’s instructions. To generate complementary DNA (cDNA), mRNA was reverse-transcribed (Superscript III; Invitrogen Life Technologies) using random primers at room temperature for 5 min, followed by 1 h at 50°C.

For quantitative PCR, cDNA was amplified using primers specific for ARAP1 isoforms and TB Green^®^ Premix Ex Taq™ II on a TP900 thermal cycler (TAKARA). The relative expression levels of ARAP1 isoforms were calculated using the comparative threshold cycle (C_T_) method and normalized to the expression level of GAPDH. ΔC_T_ values were determined by subtracting C_T(ARAP1)_ from C_T(GAPDH)_, and expression levels relative to GAPDH were defined as 2^-ΔCT^.

To detect the 33 base inserts in the ARAP1 isoforms, cDNA was amplified using primers flanking the inserts upstream and downstream. The primers used for the assays are listed below:

ARAP1isoform1-5UTR-F, TGA CTA TGA TGA TGT CCC GGA

ARAP1isoform1-R, AGC CTT GAT GAC AGG TGT GA

ARAP1isoform2-5UTR-F, CTG TGT GCT GCA GTT ACT ACC

ARAP1isoform2-R, CTG AAC TCT TCC CGG GCA

ARAP1isoform3-5UTR-F, CTC GTG GAG TTC TCT TCG CT

ARAP1isoform3-R, CCC ATG TCT AGC AGG CCA G

Arap1_Exon29F, ACA CCA AGC ATG GTA TGA TGA A

Arap1_Exon31R, TCT CGT GCT TTT CTG TCT CGT

GAPDH-Exon4F, CATGACAACTTTGGCATTGTG

GAPDH-Exon5R, GTTCAGCTCTGGGATGACCTT

### Plasmid construction of ARAP1

cDNA corresponding to isoform 5 of mouse ARAP1 mRNA (XM_006508186.3) expressed in Ba/F3 cells was amplified using KOD DNA polymerase (KOD101, TOYOBO) and cloned into pCDNA3.1-EF-neo expression vectors, in which the human cytomegalovirus (CMV) immediate-early enhancer and promoter were replaced with human EF-1α promoters (7). ARAP1 cDNA was subcloned into the CSII-EF-MCS lentiviral vector (RIKEN) fused with Venus via a linker. Deletion mutants of ARAP1 were generated using PCR and subcloned into the pCDNA3.1-EF-neo and CSII-EF-MCS vectors. Similarly, SNAP-tagged Rap1 (V12, N17) (15), RhoA (V14, N19), and Rac1 (V12, N17) constructs were amplified and cloned into the CSII-EF-MCS vectors.

### Deletion of target genes using CRISPR/Cas9 systems

Guide RNAs (gRNAs) targeting ARAP1 (at the ArfGAP and RhoGAP domains) were designed using the Cas9 Activator Tool (https://zlab.squarespace.com/guide-design-resources), cloned into pX458 vectors (#48138, Addgene), and transfected into Ba/F3 cells using the Cell Line NucleofectorTM Kit V (VVCA-1003, Lonza). GFP-positive transfected cells (0.5–1%) were sorted as single cells using an Aria III cell sorter (BD Bioscience) and were subsequently expanded. Deletion of the target genes was confirmed by the absence of the corresponding functional proteins using western blot analysis.

ARAP1 deletion in activated T cells was performed as described by Kondo et al. (16). T cells (5 × 10^6^ cells per well) from Cas9-expressing mice were activated in 24-well plates coated with anti-CD3 (8 μg/ml) and anti-CD28 (8 μg/ml) in IMDM supplemented with 4% FCS, penicillin/streptomycin, and 2-ME (T cell culture medium). After 24 h, the culture medium was removed and gently replaced with viral supernatants containing polybrene (8 μg/ml) and incubated at 37°C for 10 min. Thereafter, the plate was centrifuged at 1000 g for 1.5 h at 35°C and incubated at 37°C for 10 min. Viral supernatants were subsequently removed and replaced with T cell culture medium containing 100 U recombinant IL-2. After 24 h, the spinoculation procedure was repeated, and the viral supernatants were replaced with T cell culture medium containing IL-2. T cells were harvested and cultured after incubation at 37°C for 2 h. After 4 h, puromycin was added to a final concentration of 4 μg/ml. T cells were selected for at least 3 days and maintained in T cell culture medium containing IL-2 and puromycin until use.

The sequences of the gRNAs were as follows: GGTGATACCGCCGGGCACCA (targeting the ArfGAP domain) and GAGGGTATCTATCGAAAGTG (targeting the RhoGAP domain).

### Measurement of F-actin level

To measure the relative amount of F-actin, Ba/F3 cells (0.4 × 10^6^/100 µL) were stimulated with an equal volume of 20 nM CXCL12 (final concentration, 10 nM) in RPMI 1640 containing 10 mM HEPES and 1% fetal bovine serum (FBS) or bovine serum albumin (BSA) in 1.5 ml microtubes. At the indicated time after stimulation, the cells were fixed with three volumes of 4% paraformaldehyde (PFA) for 20–30 min and permeabilized with 1×eBioscience™ permeabilization buffer (Thermo Fisher Scientific). Thereafter, the samples were stained with iFlour647-conjugated phalloidin (1:1500). Phalloidin intensity was measured using Attune NxT flow cytometer (Invitrogen) and the relative amount of F-actin in a cell type was calculated by normalizing phalloidin intensity to the average intensity of unstimulated wild-type cells.

### Evaluation of cell polarity

Ba/F3 cells (0.4–2 × 10^6^/100 µL) were stimulated with an equal volume of 20 nM CXCL12 (final concentration, 10 nM) in RPMI 1640 containing 10 mM HEPES and 1% FBS or BSA in 1.5 microtubes. At the indicated time after stimulation, the cells were fixed with two volumes of 4% PFA for 20–30 min, permeabilized with 1×eBioscience™ permeabilization buffer (Thermo Fisher Scientific), and stained with iFlour488-conjugated phalloidin (1:5000) and APC-conjugated anti-CD44 antibodies (1:200). Images of 10,000–30,000 stained cells were acquired using the ImageStream X Mark II and analyzed with the IDEAS Application v6.1 (2). The degree of polarized F-actin distribution was quantified as the distance between the morphological center of the cell and the intensity center of phalloidin staining threshold (2).

### Western blotting

Cells were lysed with lysis buffer (1% Triton X-100, 0.1M Tris-HCl pH7.5, 0.15M NaCl, 2 mM EDTA, aprotinin, PMSF). The lysates were mixed with Laemmli sample buffer, electrophoresed, and transferred to PVDF membranes using a semi-dry transfer. Thereafter, the membranes were blocked with 5% skim milk in Tris-buffered saline (pH 7.3) containing 0.05% Tween 20 (TBST) at room temperature for 1 h and incubated with specific primary antibodies in TBST for 2–4 h at room temperature or overnight at 4°C. After several washes with TBST, the membranes were incubated with secondary antibodies in blocking buffer according to the manufacturer’s instructions. The following antibodies were used: anti-tubulin (1:3000), anti-GFP (1:5000), anti-ARAP1(1:500), anti-SNAP1 (1:1000), anti-RhoA (1:500–1000), and HRP-conjugated anti-mouse, anti-rabbit, or anti-goat IgG (1:3000–5000).

### Pull-down of Rap1 by the GST-fusion domain of ARAP1 or anti-GFP antibody

Briefly, the RA (aa 1179–1262) and RA-PH (aa 1179–1452) domains of ARAP1 were cloned into the pGEX-T3 vector, which was transformed into BL21(DE3) *E*. *coli* to generate GST-fused RA or RA-PH domains of ARAP1 (GST-RA and GST-RA-PH). Transformed BL21(DE3) cells were expanded in 200 ml of LB medium and cultured for 2 h at 32°C after induction with 0.1 mM IPTG. Thereafter, the cells were collected, sonicated to prepare lysates, and incubated with glutathione Sepharose 4B beads at 4°C for more than 2 h to capture GST-fusion proteins. 293T cells transfected with SNAP-tagged Rap1, Rap1V12, Rap1N17, Rac1V12, Rac1N17, RhoV14 and RhoN19 were lysed with lysis buffer (1% Triton X-100, 0.1M Tris-HCl pH7.5, 0.1M NaCl, 10 mM MgCl_2_, aprotinin, PMSF). Thereafter, the cell lysates were incubated with beads bound to GST-RA and GST-RAPH at 4 °C for 1 h. After washing, the beads were resuspended in 2× Laemmli sample buffer and subjected to immunoblotting.

For co-immunoprecipitation of ARAP1 and Rap1, 293T cells were transfected with Venus-ARAP1 and SNAP-Rap1 and lysed with lysis buffer (1%Triton X, 0.1M Tris-HCl pH7.5, 0.1M NaCl, 10 mM MgCl_2_, aprotinin, PMSF). Cell lysates were incubated with 2 μg anti-GFP antibodies (WAKO) for 3 h at 4 °C, and Protein-G sepharose beads were added for 1 h. After washing, the beads were suspended in 2× Laemmli sample buffer and subjected to immunoblotting.

### Measurements of RhoA activation

To perform pull-down assay for RhoA-GTP, Ba/F3 cells were washed and incubated in RPMI 1640 containing 1% fat-free BSA (Nakarai) and 10 mM HEPES at 37 °C for 4 h in a CO_2_ incubator. Thereafter, the cells (5 × 10^6^) were suspended in 50 μl of RPMI 1640 containing 10 mM HEPES and stimulated with CXCL12 in 200 ul (final concentration, 10 nM). Stimulated cells were lysed using an equal volume of 2× Lysis buffer (2% Triton X-100, 100 mM Tris-HCl pH 7.5, 300 mM NaCl, 20 mM MgCl_2_, and protein inhibitors). Glutathione Sepharose 4B beads (GE Healthcare) conjugated with GST-RalGDS-RBD were added to the cell lysates and incubated with gentle agitation at 4°C for 1 h. After two washes with lysis buffer, the beads were resuspended in 40 μl of 2× sample buffer and subjected to immunoblotting using antibodies specific to RhoA.

RhoA-GTP levels were assessed via intercellular staining using a RhoA-GTP antibody (2, 17, 18). Briefly, the cells (4 × 10^5^) were washed and incubated in RPMI 1640 containing 1% globulin-free BSA (Nakarai) and 10 mM HEPES for 1 h and stimulated with CXCL12 (final concentration, 10 nM). Thereafter, the cells were fixed with two volumes of 4% PFA, permeabilized with 1× eBioscience Permeabilization Buffer, stained with anti-RhoA (1:200) or anti-active RhoA (1:500−1000), and stained with eFluor 660 F(ab’)_2_-goat anti-mouse IgG (H+L) (1:200). The median fluorescence intensities (MFI) of RhoA and RhoA-GTP were measured using flow cytometry. the relative amount of RhoA-GTP was calculated as the ratio of RhoA-GTP MFI and of total RhoA MFI. Finally, calculated ratios were normalized to the ratio of unstimulated WT cells.

### Immunostaining for confocal microscopy

Cells were fixed with 2% PFA, washed with PBS, and plated onto poly L-coated glass-bottom dishes. Thereafter, the cells were permeabilized with a buffer containing 1% Triton X-100, 60 mM PIPES, 25 mM HEPES, and 2 mM MgCl_2_. After blocking with PBS containing 5% skim milk, the cells were incubated with anti-ARAP1 primary antibodies and bovine anti-goat IgG conjugated with Alexa488. Subsequently, the cells were stained with iFlour555-conjugated phalloidin and mounted for imaging. Z-stack images (z = 0.13 μm steps) were acquired using an Andor Dragonfly confocal microscope (Andor) with a ×100 objective lens.

### Statistical analysis

Significant differences between two groups were determined using Student’s *t*-test in Microsoft Excel. Some of the Data were analyzed using one-way analysis of variance (ANOVA) followed by Tukey’s multiple comparison test in GraphPad Prism 5, described in Figure legends. Statistical significance was set at *p* < 0.05.

## Results

### ARAP1 expression in lymphocytes

**Figure 1:**
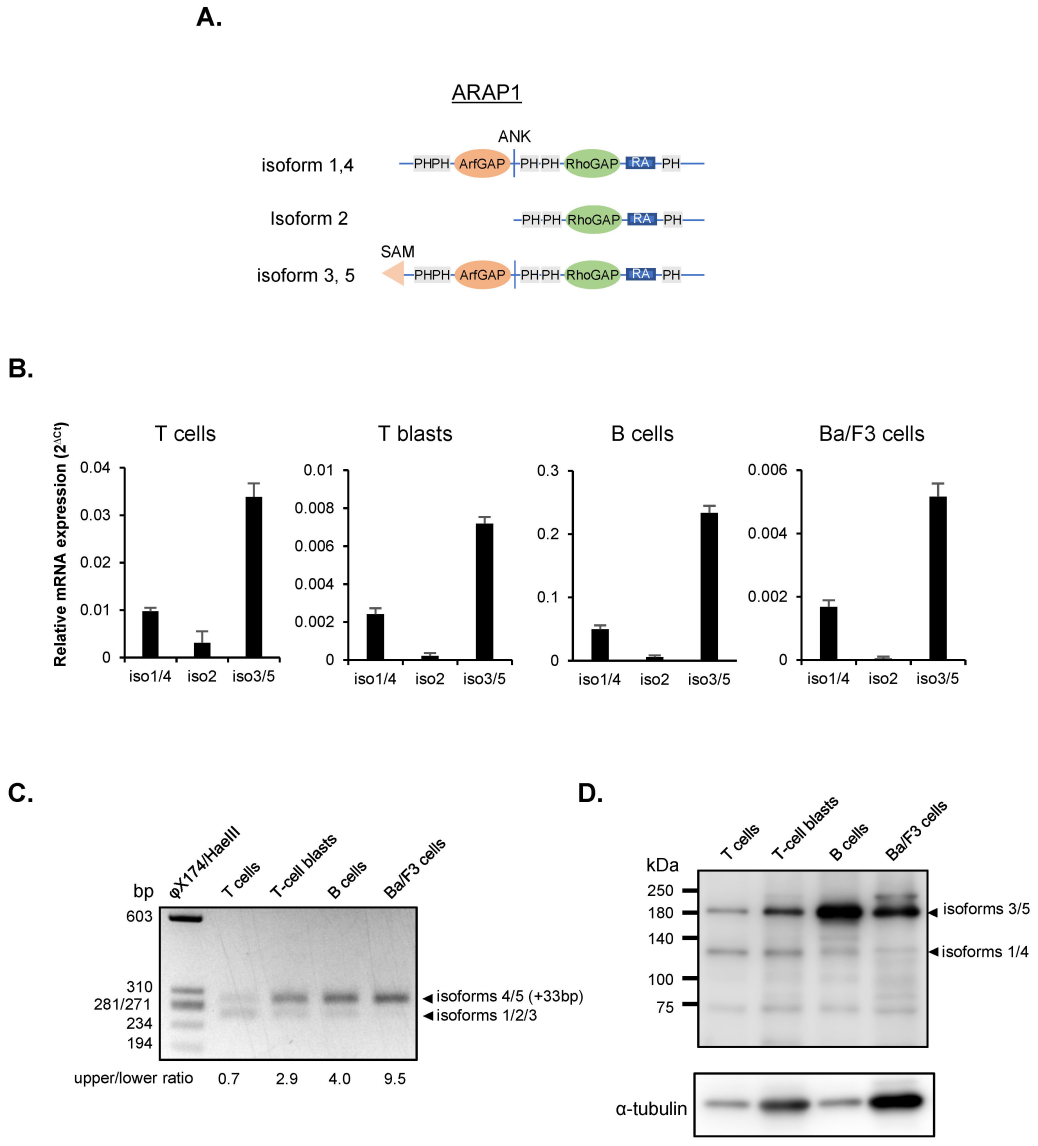
ARAP1 expression in lymphocytes. **(A)** Isoforms of ARAP1. **(B)** mRNA expression of ARAP1 isoforms (iso1/4, isoforms 1 and 4; iso2, isoform 2; iso3/5, isoforms 3 and 5) in lymphocytes, measured using quantitative RT-PCR. **(C)** The detection of 33 bp insert in PH5 domain of ARAP1 isoforms 4 and 5 using RT-PCR **(D)** ARAP1 protein expression in lymphocytes. Lysates from T cell, B cells, T-blasts and Ba/F3 cells were subjected to western blot analysis to detect ARAP protein expression.Arrowheads indicate long isoforms (~180kDa, isoforms 3 and 5) and intermediate isoforms (~130kDa, isoforms 1 and 4).

A previous study showed that Rap1 plays critical a role in the reorganization of the actin cytoskeleton in lymphocytes by controlling RhoA signaling pathways (2). However, the regulatory mechanisms underlying RhoA activation remain unclear. Among the various RhoGAP proteins, we focused on ARAP1, a dual Arf and RhoGAP protein containing an RA domain, ankyrin repeats, and a PH domain, as a potential regulator of RhoA (**Figure 1A**) (8). The full-length ARAP1 consists of a sterile alpha motif (SAM) domain, five pleckstrin-homology (PH) domains, two ankyrin repeat (ANK) domains, an ArfGAP domain and a RhoGAP domain (**Figure 1A**) (12). According to the NCBI Gene public database (Gene ID: 69710), ARAP1 has five isoforms: long isoforms 3 and 5; intermediate isoforms 1 and 4, which lack the N-terminal SAM domain; and short isoform 2, which lacks the SAM, PH1, PH2, ANK and ArfGAP domains. To examine ARAP1 mRNA expression in lymphocytes, isoform-specific quantitative RT-PCR was performed on freshly isolated T and B cells, T cell blasts and Ba/F3 cells (a pro-B cell line) using primers for isoforms 1 and 4, isoforms 3 and 5, and isoform 2 (**Figure 1B**). Among all lymphocyte subsets tested, isoforms 3 and 5 were the most strongly expressed, followed by isoforms 1 and 4, and isoform 2. Additionally, we amplified the fragment containing the additional 33 base insert in the C-terminal PH domain in isoforms 4 and 5, which distinguished them from isoforms 1 and 3 (**Figure 1C**). The result showed that freshly isolated T cells expressed the isoforms equally with and without insert, whereas T-cell blasts, freshly isolated B cells and Ba/F3 cells predominantly expressed isoforms containing the insert. In conclusion, freshly isolated T cells mainly expressed both isoforms 3 and 5, whereas T-cell blasts, freshly isolated B cells, and Ba/F3 cells predominantly expressed isoform 5 (**Figure 1C**). To examine ARAP1 protein expression in lymphocytes, we performed western blotting using anti-ARAP1 antibody (**Figure 1D**). The predominant protein expression of long isoforms (~180kDa) in lymphocytes was confirmed, except in freshly isolated T cells which exhibited similar protein expression levels between the intermediate (~130kDa) and long isoforms (~180kDa), suggesting post-transcriptional control of ARAP1 protein levels (**Figure 1D**).

### Chemokine stimulation triggers ARAP1 translocation from the cytoplasm to membrane ruffles at an early phase of polarization

**Figure 2:**
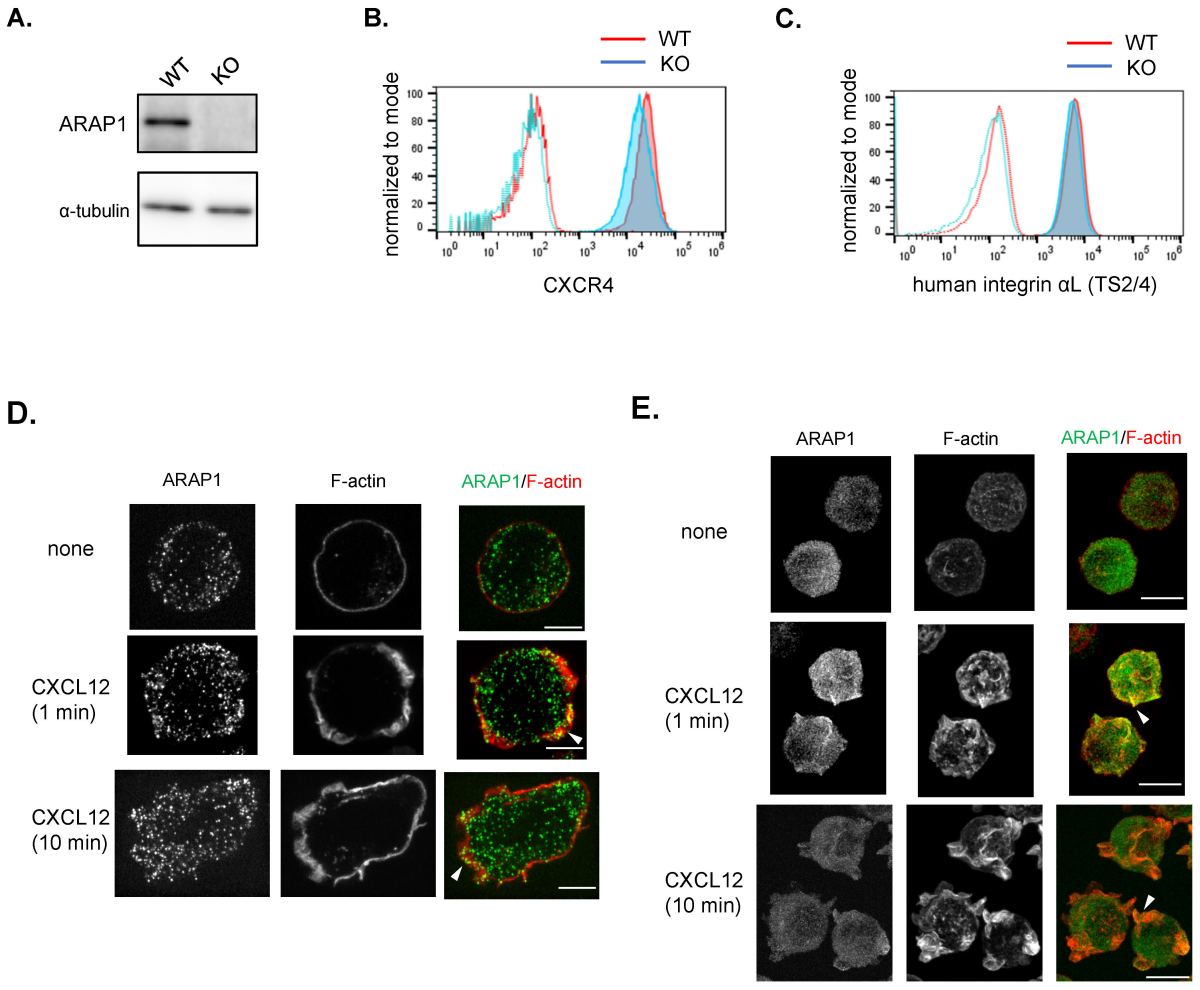
ARAP1 localization in lymphocytes after chemokine stimulation. **(A)** ARAP1 protein expression in Ba/F3.hLFA1 cells following ARAP1 knockout (KO). **(B)** CXCR4 expression of wild-type (WT) cells (red) and Arap1 knockout (KO) cells (light blue). Unstained control WT cells (dotted red) and Arap1 KO cells (dotted light blue) were also shown. **(C)** Integrin αL expression in WT cells (red) and Arap1 KO cells (light blue), measured using flow cytometry and TS2/4 antibody staining. Unstained control WT cells (dotted red) and Arap1 KO cells (dotted light blue) were also shown. **(D)** Representative Z-stack images of endogenous ARAP1 in cells unstimulated and stimulated with CXCL12 (10 nM, 1 and 10 min). ARAP1, green; F-actin: red. Arrow heads indicate ARAP1 accumulation in ruffle membranes. Scale Bar, 5 μm. **(E)** Representative Z-projection images of Venus-ARAP1 wild type in the enter body of cells unstimulated and stimulated with CXCL12 (10 nM, 1 and 10 min). ARAP1, green; F-actin, red. Scale Bar, 10 μm. Arrowheads indicate colocalization of Venus-ARAP1 and F-actin rich ruffles.

To investigate the role of ARAP1, we generated Ba/F3.hLFA1 cells lacking ARAP1 (Arap1 KO) using CRISPR/Cas9. Western blotting confirmed successful ARAP1 knockout, as evidenced by the non-detection of the protein in Arap1 KO cells (**Figure 2A**). Arap1 KO cells showed slightly reduced CXCR4 expression but maintained comparable human LFA-1 expression (**Figures 2B, C**).

In lymphocytes, chemokine rapidly induces F-actin polymerization, resulting in multiple protrusions within 1 min of the early phase, followed by F-actin polarization and lamellipodia formation in the late phage 2 min onward (2). To examine ARAP1 localization in lymphocytes upon chemokine stimulation, we performed immunostaining with anti-ARAP1 antibodies and visualized the actin cytoskeleton using fluorescently labeled phalloidin. The specificity of ARAP1 staining was confirmed by the faint signals observed in Arap1 KO cells (**Supplementary Figure S1**). In unstimulated cells, endogenous ARAP1 exhibited a punctate vesicle-like distribution in the cytoplasm (**Figure 2D**; **Supplementary Figure S1**). However, ARAP1 puncta were redistributed not only in the cytoplasm but also at multiple membrane ruffles during the early phase (1 min) and at lamellipodia during the later stage (10 min) after chemokine stimulation (**Figure 2D**; **Supplementary Figure S1**). Moreover, we investigated ARAP1 localization in Ba/F3.hLFA1 cells ectopically expressing Venus-tagged ARAP1 (Venus-ARAP1) and found that Venus-ARAP1 was mainly localized in the cytoplasm under basal conditions (**Figure 2E**). In contrast, Venus-ARAP1 rapidly accumulated at F-actin-rich ruffles within 1 min of CXCL12 stimulation (**Figure 2E**), followed by a gradual decline in ruffle localization. Collectively, these findings indicate that chemokine stimulation induces rapid translocation of ARAP1 from the cytoplasm to the membrane ruffles during the early phase of cell polarization.

### Loss of ARAP1 enhances F-actin capping and polymerization

**Figure 3:**
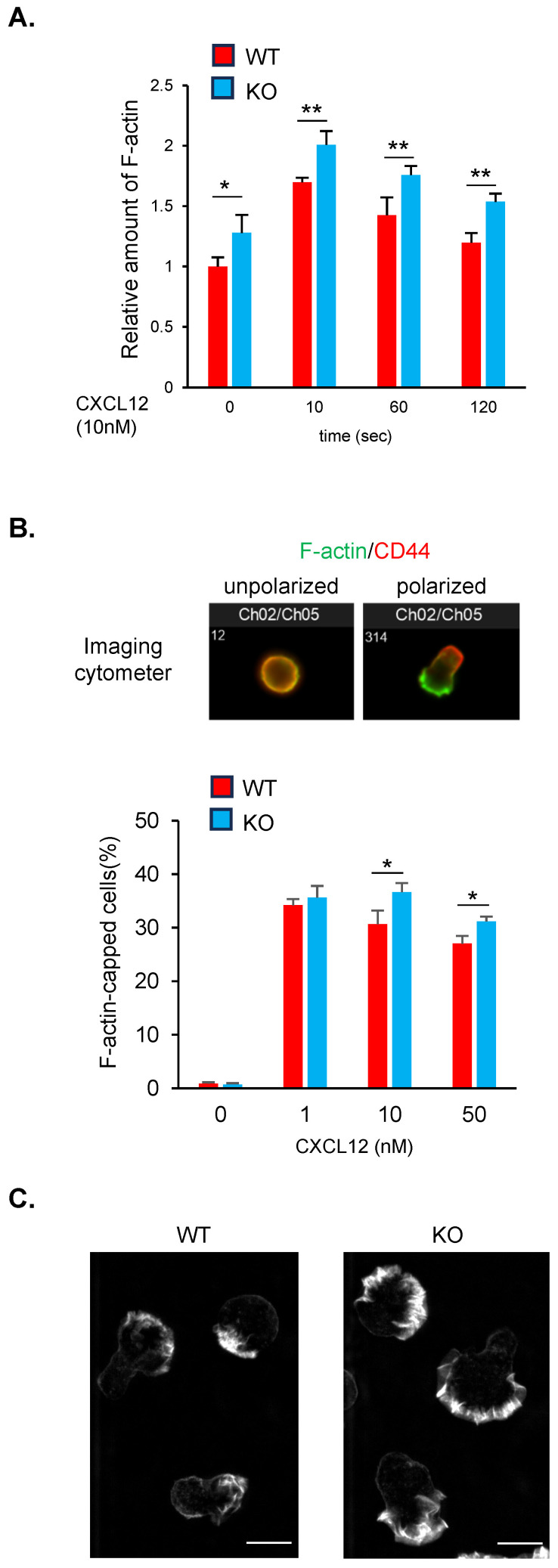
ARAP1 deficiency increased F-actin capping and polymerization in lymphocytes. **(A)** Quantification of F-actin level in WT (n = 3) and Arap1 KO (n = 3) cells following stimulation with CXCL12 (10 nM). F-actin level was visualized by staining with fluorescently labeled phalloidin and measured using flow cytometry. Median fluorescent intensity (MFI) was normalized to the average MFI of unstimulated WT cells. **(B)** The upper representative image showed F-actin and CD44 staining in unpolarized cells and chemokine-induced polarized cells (10 nM, 10 min), acquired using imaging cytometer. The lower panel shows average percentages of the F-actin capped cells among WT (n = 3) and Arap1 KO (n = 3) cells before and after CXCL12 stimulation (1, 10, 50 nM, 10 min). **(C)** Representative Z-projection images of WT and Arap1 KO cells stained with fluorescently labeled phalloidin. Scale Bar, 10 μm. Asterisks indicate statistical significance for **(A, B)** calculated using Student’s *t*-test; *p < 0.05; **p < 0.01.

Previous studies have suggested that ARAP1 regulates the actin cytoskeleton in non-hematopoietic cells (8–10, 13, 14). To investigate the role of ARAP1 in this process, we measured F-actin levels in wild-type (WT) and Arap1 KO cells after chemokine stimulation using phalloidin staining (**Figure 3A**). F-actin levels in WT cells increased rapidly by approximately 1.5-fold within 10 s of CXCL12 stimulation, followed by a gradual decrease. In contrast, Arap1 KO cells exhibited a significantly greater increase in F-actin levels in response to chemokine stimulation than WT cells (**Figure 3A**).

Chemokines induce a polarized distribution of F-actin (F-actin capping) to the leading edges (19). To assess the role of ARAP1 in this process, WT and Arap1 KO cells were stimulated with CXCL12, fixed, immunostained with fluorescently labeled phalloidin and CD44, and subjected to imaging cytometer analysis (**Figure 3B**). The degree of polarized F-actin distribution was quantified as the distance between the morphological center of the cell and the intensity center of phalloidin staining (2). Unstimulated WT and Arap1 KO cells displayed circular and unpolarized shapes (**Figure 3B**). Chemokine exposure resulted in F-actin capping in approximately 30–40% of WT cells at 10 min post-stimulation, indicating lamellipodia formation (**Figure 3B**). In contrast, Arap1 KO cells showed a higher frequency of F-actin capping than WT cells.

Furthermore, we examined F-actin distribution in Arap1 KO cells using confocal microscopy and found that WT and KO cells exhibited front-back polarity with lamellipodia at the front. However, ARAP1 KO cells showed stronger phalloidin intensity in lamellipodia than WT cells (**Figure 3C**), consistent with the results of cytometer analysis (**Figures 3A, B**). Collectively, these data indicate that ARAP1 negatively regulates F-actin capping and polymerization in response to chemokine stimulation.

### Increased ARAP1 expression inhibits RhoA activation and cell polarization via the RhoGAP and RA domain

**Figure 4:**
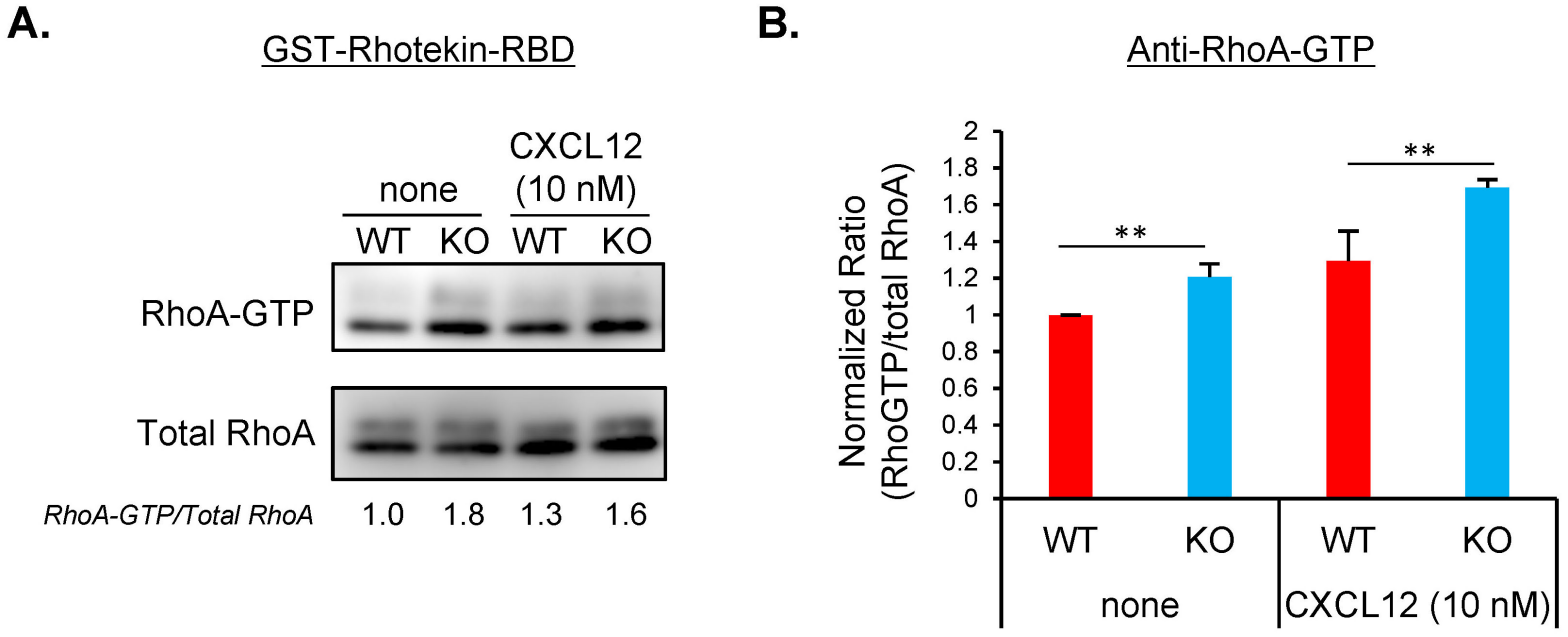
ARAP1 deficiency increased RhoA-GTP in lymphocytes. **(A)** Detection of active RhoA in WT and Arap1 KO cells using GST-Rhotekin-RBD pull-down assay. Both active (GTP-bound, the upper image) and total RhoA (the lower image) were detected using anti-RhoA antibody. The numbers under images represent relative active RhoA expression calculated by dividing the intensity of RhoA-GTP by that of total RhoA and normalizing the calculated ratios with that of unstimulated WT cells. **(B)** RhoA activation levels in WT (n = 4) and Arap1 KO cells (n = 4), measured using flowcytometry with anti-RhoA-GTP. RhoA activation levels were calculated as the intensity of RhoA-GTP staining divided by intensity of total RhoA staining. Calculated ratios were normalized to the ratio of unstimulated WT cells. Asterisks indicate statistical significance for **(A, B)** calculated using Student’s *t*-test; **p < 0.01.

Considering that ARAP1 functions through RhoGAP activity (8), we measured the levels of RhoA-GTP, the active form of RhoA, in WT and Arap1 KO cells using pull-down assays with GST-Rhotekin-RBD (**Figure 4A**). WT cells showed an increase in RhoA-GTP levels after CXCL12 stimulation compared with the basal levels in unstimulated WT cells. However, Arap1 KO cells exhibited higher RhoA-GTP levels than WT cells, both in the absence and presence of CXCL12. Similar results were obtained when RhoA activation was assessed using an anti-RhoA-GTP antibody (**Figure 4B**) (2, 17, 18). Overall, these findings are consistent with the function of ARAP1 as a RhoGAP.

**Figure 5:**
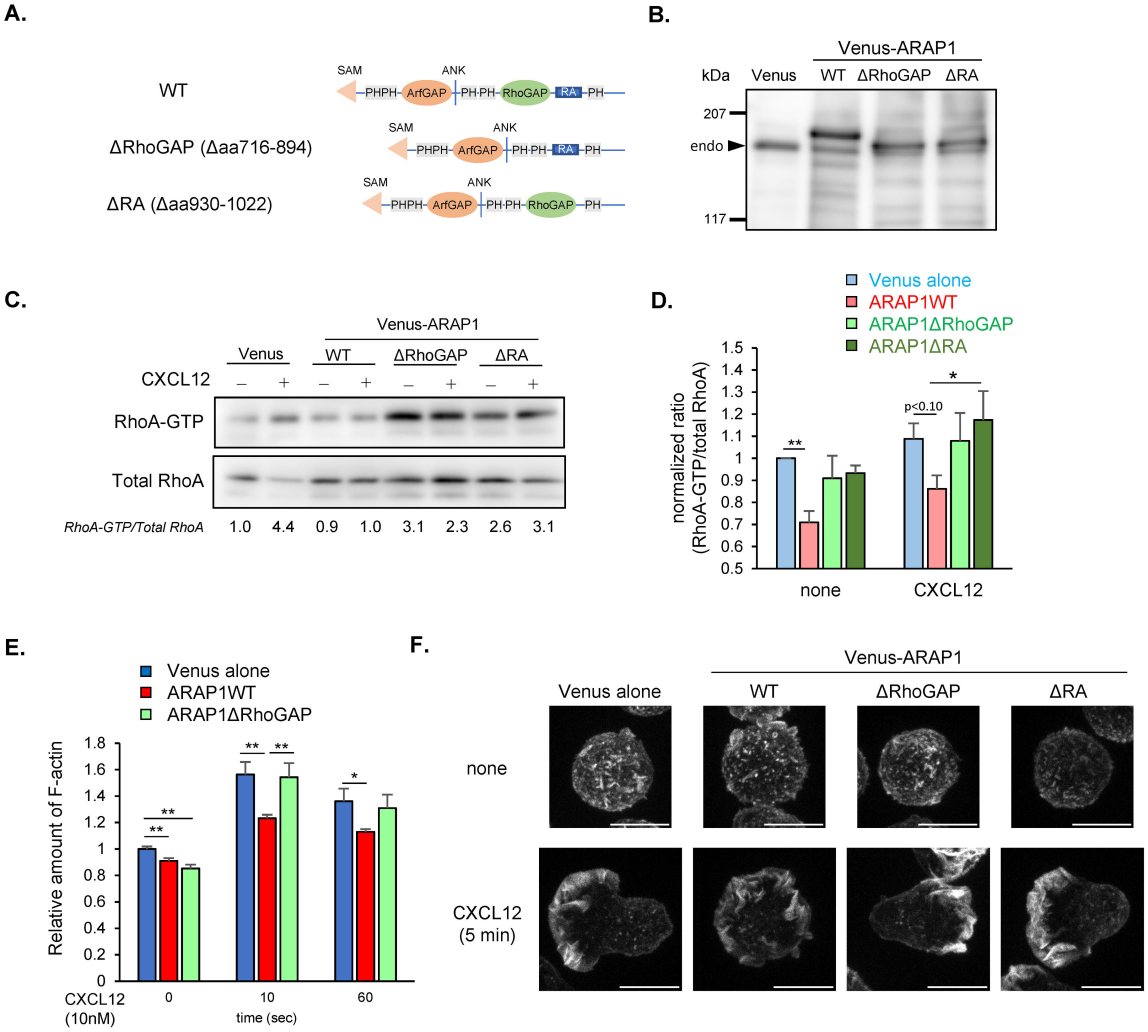
Ectopic ARAP1 expression inhibits RhoA activation via RhoGAP and the RA domain. **(A)** Schematic diagram of ARAP1 mutants with RhoGAP domain (ΔRhoGAP) or RA domain (ΔRA) deletion. **(B)** Expression of Venus alone, Venus-ARAP1 wild-type (WT), Venus-ARAP1ΔRhoGAP, and Venus-ARAP1ΔRA in Ba/F3 transfectants, detected using western blotting. The arrowhead shows endogenous ARAP1. **(C)** Detection of active RhoA in cells expressing Venus alone or Venus-Arap1 mutants using GST-Rhotekin-RBD pull-down assay. Both active (GTP-bound, the upper image) and total RhoA (the lower image) were detected using anti-RhoA antibody. The numbers under images represent relative active RhoA expression calculated by dividing the intensity of RhoA-GTP by that of total RhoA and normalizing the calculated ratios to that of unstimulated Venus-transfected cells. **(D)** RhoA activation levels in Ba/F3 transfectants expressing Venus alone (n = 3), Venus-ARAP1WT (n = 3), Venus-ARAP1ΔRhoGAP (n = 3), and Venus-ARAP1ΔRA (n = 3) mutants with and without CXCL12 stimulation (10 nM, 5 min) measured using flowcytometry with anti-RhoA-GTP and anti-total RhoA staining. RhoA activation levels were calculated as the intensity of RhoA-GTP staining divided by intensity of total RhoA staining. The calculated ratios were then normalized to the average ratio of unstimulated Venus-transfected cells. **(E)** Quantification of F-actin level in cells expressing Venus (n = 3), Venus-ARAP1WT (n = 3), and Venus-ARAP1ΔRhoGAP (n = 3) mutant following stimulation with CXCL12 (10 nM) using fluorescently labeled phalloidin staining. Percentages of polarized cells are shown. **(F)** Confocal imaging of cells expressing ARAP1 mutants stained with fluorescently labeled phalloidin. Scale bars, 10 µm. Statistical analysis for **(D, E)** was performed using one-way ANOVA with Tukey’s multiple comparison test. Asterisks indicate statistical significance; *p < 0.05, **p < 0.01.

To determine whether RhoGAP activity is required for ARAP1-mediated inhibition of RhoA activation, we generated Ba/F3 cells expressing a mutant ARAP1 lacking the RhoGAP domain (Venus-tagged ARAP1ΔRhoGAP, **Figures 5A, B**) and measured RhoA-GTP levels using pull-down assays. Ectopic expression of Venus-Arap1 did not alter basal RhoA-GTP levels but strongly suppressed the CXCL12-induced increase (**Figure 5C**). In contrast, RhoA-GTP levels in cells expressing ARAP1ΔRhoGAP were comparable to those in chemokine-stimulated control cells expressing Venus, regardless of CXCL12 stimulation. Moreover, the basal increase in active RhoA levels after ARAP1ΔRhoGAP overexpression could reflect its dominant negative effect, as a similar increase was observed following loss of ARAP1 (**Figure 4A**). Similar results were obtained using anti-RhoA-GTP staining, except for a minimal increase after CXCL12 stimulation (**Figure 5D**), which was likely due to differences in resting conditions of the cells (see Methods). Collectively, these results suggest that ARAP1 inhibits RhoA activation via its RhoGAP domain.

To test whether ARAP1 regulates F-actin polymerization via RhoGAP activity, we measured F-actin levels in WT cells and transfectants expressing Venus-ARAP1 or ARAP1ΔRhoGAP after chemokine stimulation using flow cytometry analysis with phalloidin staining. Ectopic ARAP1 expression reduced F-actin levels after CXCL12 stimulation compared with those in control cells (**Figure 5E**). However, this reduction was rescued by deletion of the RhoGAP domain, suggesting that ARAP1 suppresses F-actin polymerization via RhoA inhibition (**Figure 5E**). Overall, these data indicate that ARAP1 inhibits RhoA-mediated F-actin polymerization through its RhoGAP domain.

To further assess the role of ARAP1 RhoGAP activity in cell polarization, the transfectants were stimulated with CXCL12, fixed, immunostained with fluorescently labeled phalloidin, and analyzed using confocal microscopy (**Figure 5F**). Cells transfected with Venus alone exhibited a polarized morphology. In contrast, ARAP1 WT transfectants showed a profound defect in cell polarization, characterized by multiple ruffles. Deletion of the RhoGAP domain rescued this defect, indicating that ARAP1 inhibits cell polarization via its RhoGAP activity.

The Ras-associated (RA) domain of ARAP1 has been reported to affect ARAP1 function in fibroblasts (10). To investigate the impact of RA-domain deletion in ARAP1-mediated RhoA inhibition in lymphocytes, we generated cells expressing a Venus-tagged ARAP1 mutant lacking the RA domain (ARAP1ΔRA, **Figures 5A, B**). Thereafter, we measured RhoA-GTP levels in Venus-ARAP1ΔRA transfectants using RhoA pull-down assays with GST-Rhotekin RBD (**Figure 5C**). In contrast to the decreased RhoA-GTP levels observed in cells expressing wild-type ARAP1, cells expressing Venus-ARAP1ΔRA displayed RhoA-GTP levels comparable to those of Venus-transfected controls after chemokine stimulation, indicating that RA domain deletion abolished the inhibitory effect of ARAP1 on RhoA-GTP levels (**Figure 5C**). Additionally, the basal RhoA-GTP levels were higher in unstimulated Venus-ARAP1ΔRA-expressing cells than in unstimulated Venus-transfected control cells (**Figure 5C**). Immunostaining with RhoA-GTP antibodies also revealed similar results (**Figure 5D**). To assess the role of the RA domain in cell polarization, we compared F-actin distribution in ARAP1ΔRA-transfected cells and Venus-ARAP1-transfected cells (**Figure 5F**). Confocal imaging revealed that the polarization defect observed in Venus-ARAP1-expressing cells was restored in ARAP1ΔRA-expressing cells. Overall, these findings indicate that ARAP1 requires the RA domain to inhibit RhoA activation.

### RA domain of ARAP1 binds to Rap1

**Figure 6:**
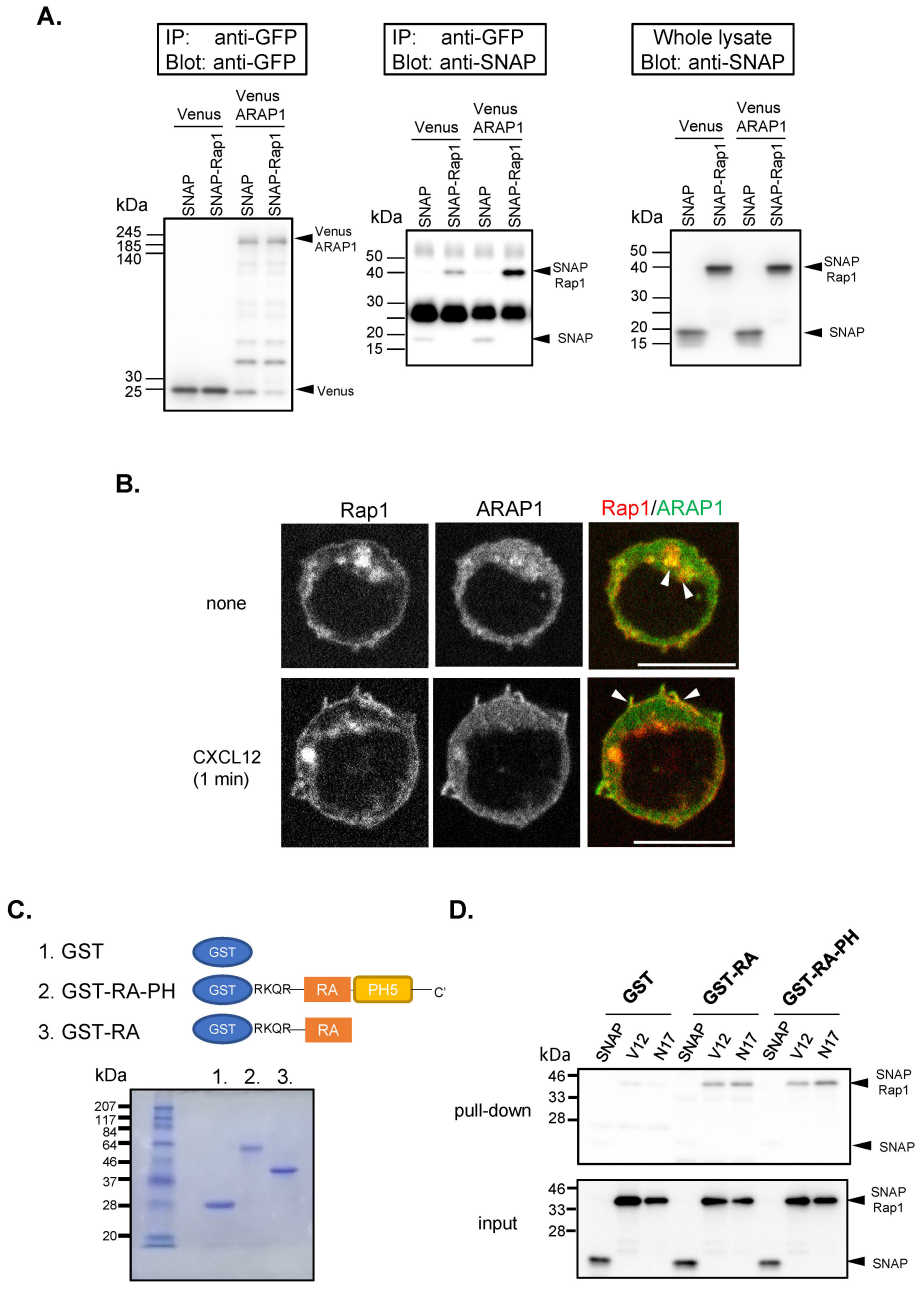
ARAP1-RA domain bound to Rap1. **(A)** Co-immunoprecipitation assay of Venus-ARAP1 and SNAP-Rap1 using anti-GFP antibody. The left panel shows precipitated Venus alone and Venus-Arap1; the middle panel shows co-immunoprecipitated SNAP alone and SNAP-Rap1; the right panel shows the inputs of SNAP and SNAP-Rap1 from whole lysates. **(B)** The distribution of Venus-ARAP1 and Turquoise2-Rap1 in unstimulated and CXCL12-stimulated (CXCL12, 10 nM, 1 min) cells. Confocal images of Venus-ARAP1 and Turquoise2-Rap1 and the merged images are shown. Arrowheads represent the location where ARAP1 and Rap1 colocalize. Scale bar, 10 µm **(C)** Schematic diagram of GST, GST-RA, and GST-RA-PH5-C-terminal domains. The image shows Coomassie staining of these proteins enriched using Glutathione Sepharose beads. **(D)** RA domain of ARAP1 bound to Rap1. Lysates from 293T cells transfected with SNAP-Rap1 mutants were subjected to pull-down assay using GST, GST-RA, and GST-RA-PH5-C-terminal domain. Representative images of pull-down samples (upper image) and their inputs (lower image) are shown.

A previous study showed that ARAP3 binds to Rap1 through its RA domain (20). To examine whether ARAP1 binds to Rap1, we performed a co-immunoprecipitation assay using full-length Venus-ARAP1 and SNAP-tagged wild-type Rap1 expressed in 293T cells (**Figure 6A**). Rap1 co-precipitated with full-length ARAP1 but little with Venus alone, indicating that ARAP1 can bind to Rap1.

To investigate whether ARAP1 colocalizes with Rap1 in lymphocytes, we obtained confocal images of Ba/F3 cells transfected with Venus-ARAP1 and Turquoise2-tagged Rap1(TQ2-Rap1) before and after CXCL12 stimulation (**Figure 6B**). Before stimulation, Venus-ARAP1 expression was mainly localized to the cytoplasm, with a fraction localized to vesicle-like structures. TQ2-Rap1 was mainly localized to vesicular-like structures, where it colocalized with Venus-ARAP1 (**Figure 6B**). However, a fraction of Venus-ARAP1 was translocated to the ruffle membrane with increased F-actin intensity within 1 min of CXCL12 stimulation. Simultaneously, TQ2-Rap1 also translocated to the membrane and colocalized with Venus ARAP1 (**Figure 6B**).

To determine whether the RA domain of ARAP1 binds to Rap1, we performed pull-down assays using GST-fused RA to capture SNAP-tagged Rap1 mutants expressed in 293T cells (**Figure 6C**). Some RA domains, such as those in RIAM, require a PH domain for efficient Rap1 binding (21). Therefore, we also used GST-fused RA with a C-terminal PH-domain module (GST-RA-PH) (**Figure 6C**). Both GST-RA and GST-RA-PH pulled down both the active (RapV12) and inactive (RapN17) forms of Rap1, whereas GST alone did not (**Figure 6D**). Collectively, these results suggest that the RA domain alone can bind to Rap1. Additionally, we examined whether the RA domain of ARAP1 binds to the Rho GTPases Rac1 and RhoA using pull-down assay (**Supplementary Figure S2**). The ARAP1-RA domain bound both active (RacV12) and inactive (RacN17) forms of Rac1 and the inactive form of RhoA (RhoN19). Overall, these data suggest that ARAP1 constitutively interacts with Rap1 and Rac1 via its RA domain.

### The role of Arap1 in chemokine-induced lymphocyte migration

**Figure 7:**
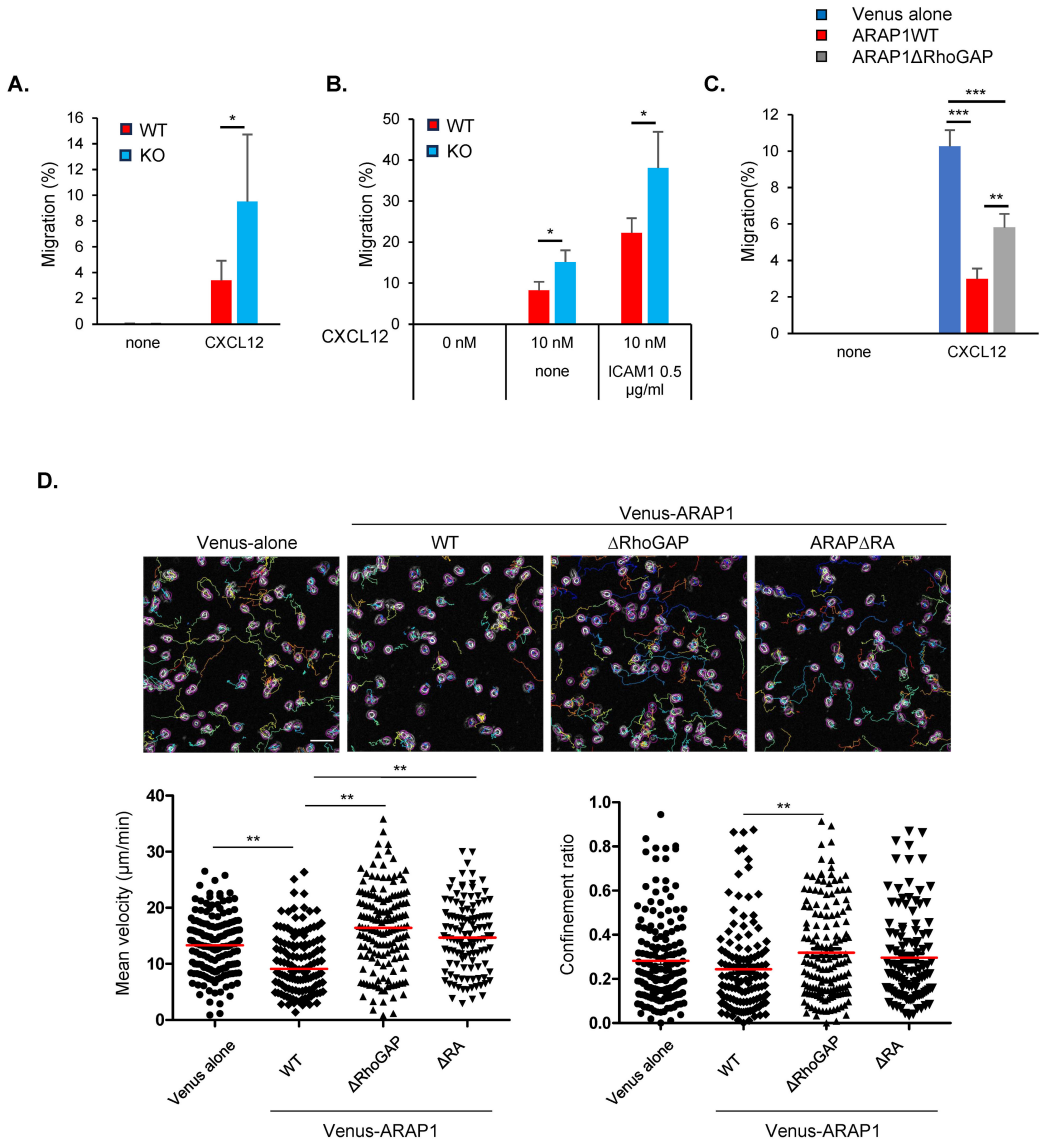
ARAP1 inhibited lymphocyte migration. **(A)** Chemotactic migration efficiency of WT and Arap1KO T cells. Cells (1 × 10^5^) were loaded onto upper chamber and allowed to migrate to lower chamber for 3 h The lower chambers contained CXCL12 at concentrations of 0 (n = 5), 10 nM (n = 5). **(B)** Chemotactic migration efficiency of WT and Arap1KO T cells on integrin-ligand ICAM-1. The upper chambers were coated with 100 μg/ml of anti-human IgG capture antibody, followed by loading of 0 or 0.5 μg/ml of human ICAM1. The lower chambers contained CXCL12 at concentrations of 0 (n = 3), 10 nM (n = 3). **(C)** Migration efficiency of Ba/F3.hLFA1 cells expressing ARAP1 mutants towards CXCL12 using transwell migration assay. The lower chambers contained CXCL12 at concentrations of 0 (n = 1), 3 nM (n = 3). **(D)** Migration of Ba/F3.hLFA1 cells expressing ARAP1 mutants on immobilized ICAM-1 (0.5 μg/ml) and CXCL12 (50 nM). Upper images display individual cell tracks. Scale bar, 50 µm. The lower left panel showed track velocity (μm/min); the lower right panel confinement ratio calculated as the displacement divided by total path length. Statistical analysis was performed using Student’s *t*-test for **(A, B)** and using one-way ANOVA with Tukey’s multiple comparison test for **(C, D)** Asterisks indicate statistical significance; *p < 0.05, **p < 0.01, ***p < 0.001.

Given the importance of RhoA regulation in lymphocyte migration, we examined the role of ARAP1 in this process. Specifically, we measured the chemotactic migration of Arap1 KO cells with or without ICAM-1, a ligand of integrin LFA-1, using a transwell migration assay. Compared with WT T cells, Arap1 KO cells exhibited enhanced migration to 10 nM CXCL12 in the lower chamber (**Figure 7A**). Coating the upper chamber with ICAM-1, which serves as an adhesion substrate, promoted the migration of WT cells (**Figure 7B**), consistent with the role of LFA-1 in chemokine-mediated lymphocyte migration (22). Arap1 KO cells migrated more strongly toward 10 nM CXCL12 than WT cells in the presence of ICAM-1 (**Figure 7B**). Thus, loss of ARAP1 enhanced chemokine-mediated cell migration.

To investigate the effect of ARAP1 and the role of its RhoGAP domains in chemotactic migration, we performed transmigration assays using cells expressing different ARAP1 mutants. ARAP1 overexpression inhibited migration toward CXCL12 (**Figure 7C**). Deletion of the RhoGAP domain partially restored the migration defect caused by ARAP1 overexpression (**Figure 7C**). The residual defect could be due to ArfGAP activity, which regulates F-actin dynamics (8–10). Overall, these results suggest that ARAP1 negatively regulates lymphocyte migration by inhibiting RhoA through its RhoGAP domain.

To further assess the role of ARAP1 and its RhoGAP and RA domains in cell migration on an integrin ligand, we tracked the migration of cells expressing ARAP1 mutants on immobilized ICAM-1 and CXCL12 in a glass-bottom dish using time-lapse imaging and measured the migration speed and confinement ratio (displacement divided by distance, representing migration directionality) (**Figure 7D**). Ectopic expression of Venus-ARAP1 significantly reduced the migration speed, with minimal effect on the confinement ratio. Deletion of the RhoGAP and RA domains rescued the migration defect. Overall, these results indicate that ARAP1 controls migration speed via RhoGAP activity and Rap1.

To investigate the role of ARAP1 in primary T cells, we generated T-cell blasts with defective ARAP1 expression using T cells from Cas9 (hSpCas9) knock-in (Cas9-KI) mice and retroviral *Arap1* gRNA (23, 24). ARAP1 protein downregulation in T cells was confirmed by western blotting (**Supplementary Figure S3A**). To assess RhoA activation in Arap1 KO cells, we stained control and Arap1 KO T cells with anti-RhoA-GTP antibodies and found that RhoA-GTP levels were higher in Arap1 KO T cells than in control cells after CCL21 stimulation (**Supplementary Figure S3B**), consistent with the RhoGAP activity of ARAP1. To examine the effect of ARAP1 on T cell polarization, we measured F-actin intensity and accumulation in control and Arap1 KO cells after CCL21 stimulation using an imaging cytometer (**Supplementary Figure S3C**). Arap1 KO T cells exhibited higher F-actin intensity and a trend toward increased F-actin accumulation (*p* = 0.07) at 10 min after CCL21 stimulation.

To investigate the role of ARAP1 in T cell migration, we assessed chemotactic migration toward CXCL12 and CCL21 using a transwell migration assay (**Supplementary Figures S3D, E**). The migration efficiency of Arap1 KO T cells was comparable to that of the control T cells. Collectively, these observations suggest that ARAP1 plays a regulatory role in RhoA activation in T cells but is dispensable for their migration. Other Rho regulators may compensate for ARAP1 deficiency in activated T cells.

## Discussion

In this study, we demonstrated that ARAP1 functions as a novel negative regulator of RhoA activation and F-actin polymerization. ARAP1 was localizes to both the cytoplasm and membrane ruffles and inhibits RhoA activation throughout the cell via its RhoGAP domain. Moreover, the RA domain interacts with Rap1 and Rac1 and is required for the RhoGAP activity of ARAP1. ARAP1 may ensure optimal migratory responses in lymphocytes by suppressing RhoA-dependent F-actin polymerization at Rac/Rap1 rich cell protrusions.

Rac, CDC42, and RhoA (Rho family members) play central roles in F-actin polymerization following chemokine stimulation. Rac and CDC42 activate WASP/WAVE2 and the Arp2/3 complex to generate branched actin filaments, whereas RhoA activates mDia and profilin to facilitate linear F-actin polymerization. Chemokines initially cause a burst of F-actin polymerization by Rac1, leading to the formation of multiple ruffles, which in turn activates Rap1 via the F-actin scaffold, triggering the activation of Rho signaling via GEF-H1 and talin1 (2). RhoA and talin1 cooperatively activate MLC to drive actomyosin contractions (2). Additionally, RhoA activates mDia to support linear F-actin polymerization, contributing to lamellipodia formation at the leading edge. The RhoA-mDia axis is essential for F-actin polymerization, as chemokine-induced F-actin polymerization and migration are impaired in mDia1 knockout T cells (5). In this process, we found that ARAP1 inhibited both basal and chemokine-induced RhoA activation and F-actin polymerization via its RhoGAP domain. ARAP1 rapidly localizes to membrane ruffles after chemokine stimulation, which may limit RhoA-mediated F-actin polymerization through its RhoGAP activity (5, 25). Therefore, Arap1-mediated RhoA deactivation may restrain excessive F-actin polymerization, thereby suppressing lamellipodia formation and inhibiting lymphocyte migration.

The RA domain of ARAP1 binds Rap1, Rac1, and the inactive form of RhoA, and is required for RhoGAP activity and function. Consistent with our observations, a previous study showed that the RA domain of ARAP3, another member of the ARAP protein family, binds to Rap1 and promotes its RhoGAP activity and F-actin regulation (20). Both Rap1 and Rac1 accumulate at the sites of F-actin polymerization after chemokine stimulation (2, 26). Thus, the binding of the RA domain to Rap1/Rac1 could allow ARAP1 to inhibit RhoA activation and regulate the level of F-actin polymerization at these sites. Given that Rap1 is required for RhoA activation (2), it exerts both positive and negative controls over RhoA activity.

In T cells, ARAP1 knockout increased RhoA activation and F-action polymerization but was not required for cell migration. Several studies have reported that other Rho inhibitors, such as Myo9b and Fam65B, are expressed in T cells and suppress RhoA activation and migration (17, 18). These inhibitors may compensate for the loss of ARAP1 and mask its effects. Consequently, the phenotypic outcome of ARAP1 deficiency may vary depending on the cellular context and the balance between positive and negative regulators of Rho. Our study highlights the robustness and redundancy of Rho regulatory networks in primary T cells and underscores the importance of identifying the unique RA domain–dependent contributions of ARAP1. Future studies on this redundancy will further expand the physiological relevance of our findings.

In conclusion, we identified another F-actin regulatory pathway in which ARAP1 optimizes RhoA activity via its RhoGAP and RA domains to inhibit F-actin polymerization. ARAP1 may contribute to the precise translation of chemokine cues within the immune environment and fine-tune migration during immune responses.

## Data Availability

The original contributions presented in the study are included in the article/**Supplementary Material**. Further inquiries can be directed to the corresponding author.
