# Supplementary material for: ARAP1 fine-tunes F-actin polymerization level in lymphocytes through RhoA inhibition
Source: Front Immunol. 2025 Dec 18;16:1591450. doi: 10.3389/fimmu.2025.1591450 (PMC12756398; doi:10.3389/fimmu.2025.1591450)
Supplement: Supplementary file 1 [file DataSheet1.pdf]

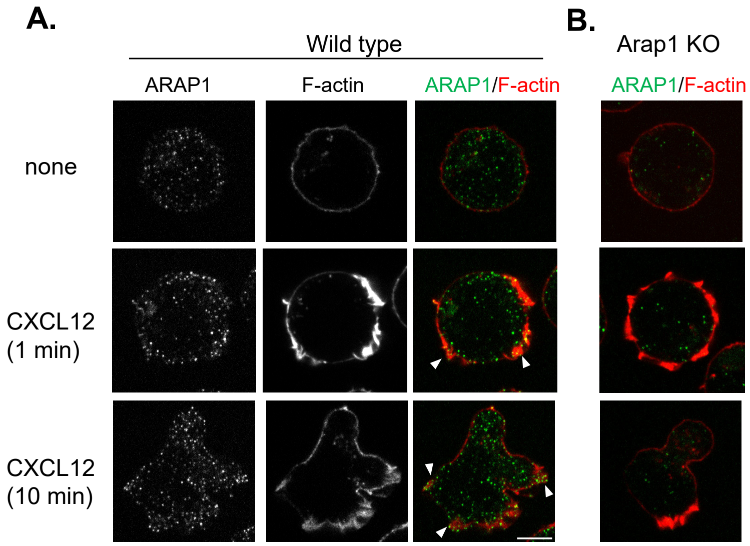

Figure S1 ARAP1 localization upon chemokine stimulation in lymphocytes.
(A) Representative images (a single plane) of endogenous ARAP1 in cells unstimulated and stimulated with CXCL12 (10 nM, 1 and 10 min). ARAP1, green; F-actin, red. Arrow heads indicate the accumulation of ARAP1 in ruffle membranes. Scale Bar, 10 µm. (B) Representative images of ARAP1 staining in Arap1 KOcells unstimulated and stimulated with CXCL12 (10 nM, 1 and 10 min).

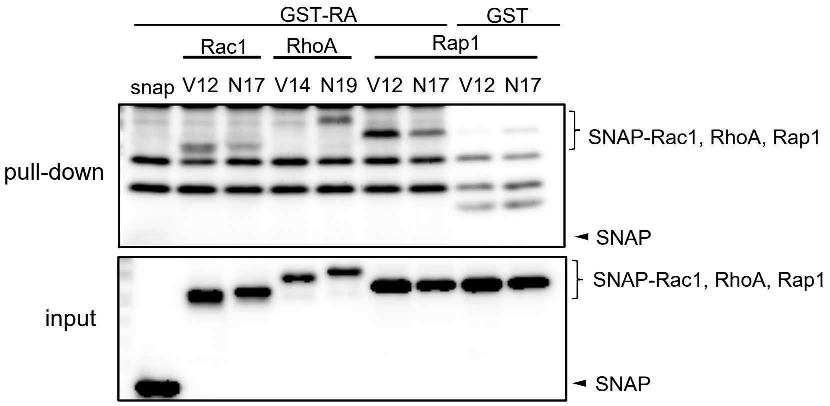

Figure S2 ARAP1 bound to Rap1, Rac1 and inactive RhoA
RA domain of ARAP1 binds to Rap1, Rac1, and inactive RhoA. Lysates from 293T cells transfected with SNAP-Rap1, Rac1, and RhoA mutants were subjected to pull-down assay using GST, and GST-RA of ARAP1. Representative blots of pull-down samples (upper image) and their inputs (lower image) are shown.

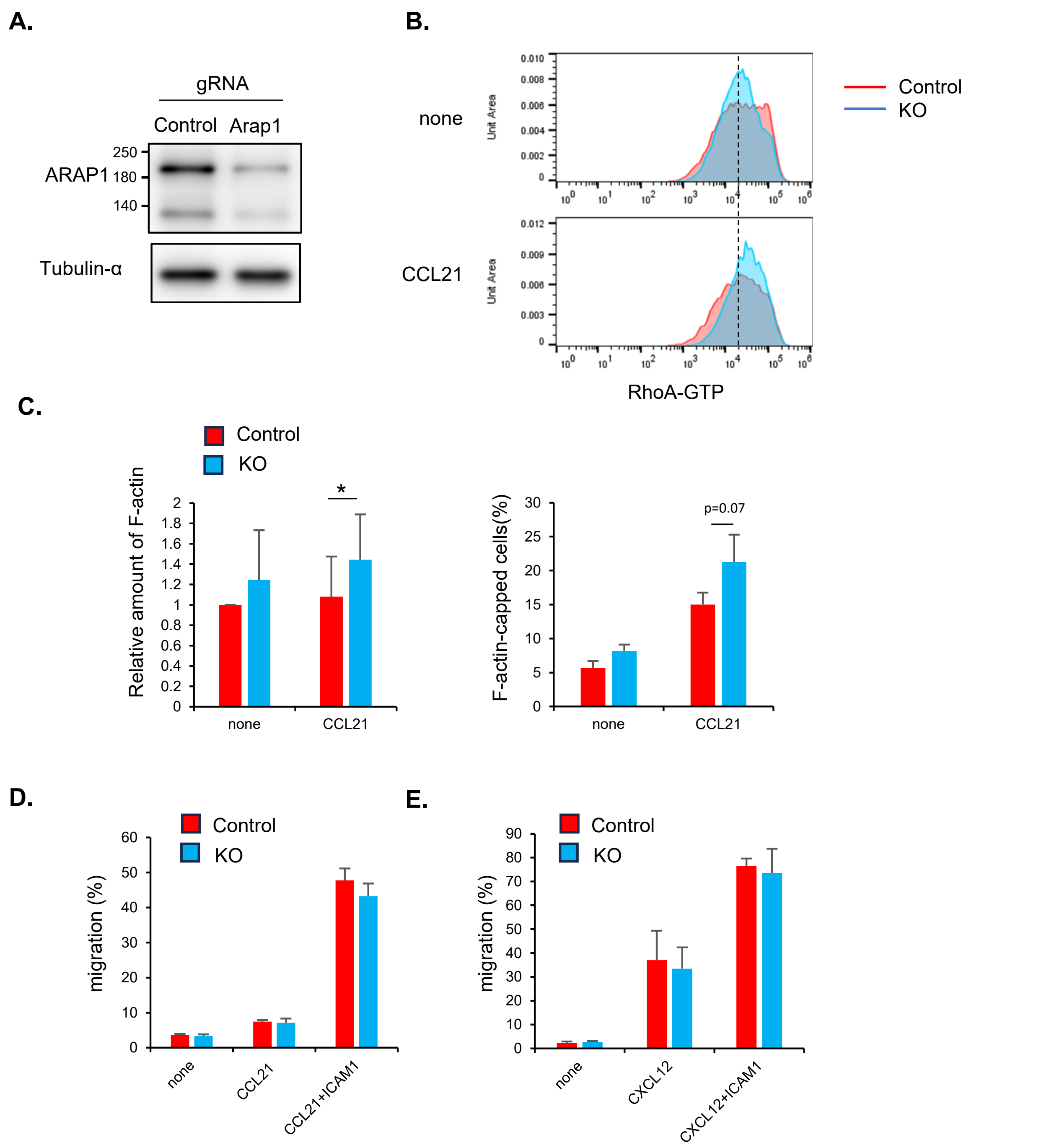

Figure S3 The effect of ARAP1 deletion in activated T cells

(A) ARAP1 protein expression in activated Cas9-knock-in T cells transfected with control and Arap1 retroviral gRNA. Cells transfected with ARAP1 gRNA exhibited approximately 70% reduction in ARAP1 protein level compared with those transfected with control gRNA. (B)The histogram shows anti-RhoA-GTP staining intensity in control and Arap1 knockout (KO) T cells with or without CCL21 stimulation, measured using flow cytometry. (C) The left panel shows F-actin level in WT (n = 3) and Arap1 KO (n = 3) T cells. Right panel shows the frequency of F-actin capped cells among WT (n = 3) and Arap1 KO (n = 3) T cells. (D, E) Chemotactic migration efficiency of WT (n = 3) and Arap1KO (n = 3) T cells on integrin-ligand ICAM-1. The upper chambers were coated with 30 μg/ml of anti-human IgG capture antibody, followed by loading of 0 or 0.5 μg/ml of human ICAM-1. The lower chambers contained CCL21 at concentrations of 0 (n = 3) and 30 nM (D) or CXCL12 at concentrations of 0 and 10 nM (E). Statistical significance was analyzed using Student's t-test; Asterisks indicate statistical significance; *, $p < 0.05$.
